# Identification of anti-fibrotic compounds from *Piper longum L* via hollow fiber cell fishing and high-performance liquid chromatography with in vivo and in vitro validation

**DOI:** 10.1186/s13020-025-01177-z

**Published:** 2025-08-19

**Authors:** Jie Tian, Nan Zhou, Heng Wang, Wanghui Jing, Guoping Zheng

**Affiliations:** 1 Department of Biochemistry and Molecular Biology, School of Basic Medical Science, Shanxi Medical University, Taiyuan, Shanxi China; 2 Department of Pharmacy, First Hospital of Shanxi Medical University, Taiyuan, Shanxi China; 3 Centre for Transplant and Renal Research, Westmead Institute for Medical Research, The University of Sydney, Sydney, NSW Australia; 4 Shaanxi Engineering Research Center of Cardiovascular Drugs Screening & Analysis, Xi’an, Shanxi China; 5 School of Pharmacy, Health Science Center, Xi’an Jiaotong University, Xi’an, Shanxi China

**Keywords:** *Piper longum* L, A hollow fiber cell fishing, Renal fibrosis, Activity screening, HPLC

## Abstract

**Background:**

Traditional Chinese medicine (TCM) is an important source of bioactive compounds, hence enjoying a wide application in clinical treatment, while its pharmacodynamic material basis remains difficult to elucidate as it has a complex chemical composition. *Piper longum* L is a commonly used herbal medicine in prescriptions for chronic kidney disease (CKD), yet its key active ingredients responsible for anti-renal fibrosis effects remain unclear. This study aimed to establish a novel and efficient strategy for the screening and identification of anti-fibrotic compounds from *Piper longum* L.

**Methods:**

HK-2 cells with fibrotic features induced by transforming growth factor-β (TGF-β) were used to develop a hollow fiber cell fishing coupled with high-performance liquid chromatography (HFCF-HPLC) mode. This model was integrated with network pharmacology and molecular biology techniques to screen and validate active anti-fibrotic compounds in *Piper longum* L. The in vivo efficacy of the identified compounds was further evaluated by a unilateral ischemia–reperfusion injury (uIRI) model with delayed contralateral nephrectomy in C57BL/6 J mice. Serum creatinine (SCr) and blood urea nitrogen (BUN) levels were taken into account to assess the renal function, while immunohistochemistry and western blot served for analyzing the fibrosis markers α-SMA and collagen-I.

**Results:**

HFCF-HPLC screening identified two key active compounds from *Piper longum* L: piperlongumine (PIPA) and piperlonguminine (PLG). Among them, PIPA exhibited the strongest inhibitory effect on the expression of fibrosis markers in vitro. In vivo studies demonstrated that PIPA significantly reduced renal fibrosis in the uIRI model, as indicated by lower SCr and BUN levels, improved renal histopathology, and reduced extracellular matrix deposition.

**Conclusions:**

A novel HFCF-HPLC model was successfully established to screen active compounds from TCM against renal fibrosis. PIPA was identified as a promising anti-fibrotic agent from *Piper longum* L, demonstrating significant renoprotective effects in in vitro and in vivo models. This work advances the modernization of herbal medicine research by offering a integrated strategy for identifying bioactive TCM components.

**Supplementary Information:**

The online version contains supplementary material available at 10.1186/s13020-025-01177-z.

## Introduction

Traditional Chinese medicine (TCM) is an essential source of drugs with unique advantages of multiple pharmacological components, and is commonly used to clinically treating human diseases in China. From 1981 to 2019, the FDA has approved 441 natural products and their derivatives for treatment of human diseases, including cancer, inflammation, cardiovascular disease and so on [1]. However, the complex chemical components of TCM increasing difficulty in elucidating its pharmacodynamic material basis. The conventional methods for analyzing TCM active components are slow and laborious. They are usually based on natural medicinal chemistry/component isolation, serum medicinal chemistry, serum pharmacology, metabolomics and molecular recognition techniques [2–8]. The network pharmacology [9], provides a new platform for analysis of Chinese herbal medicine by synthesizing bioinformatics, chemoinformatics and medical informatics.

Modern pharmacology and pharmacokinetics suggest that the interactions between drugs and their targets on the cells, are critical in determining their pharmacological effects [10, 11]. Based on the above theory, significant advancement has been made on targeted bionic screening method. Especially cell membrane chromatography (CMC), proposed by Langchong, He in 1996 [12], has been shown to be a trustworthy tool for identifying pharmacological components that act on cell membrane receptors. Recently, a stop-flow comprehensive 2D HK-2 and HK-2/CIKI cell membrane chromatography comparative analysis system screened active ingredients from Pyrrosia calvata (Bak.) Ching against crystal-induced kidney injury [13]. According to the interactions between active components and living cells [14, 15], we constructed a HFCF-HPLC model to screen major anticancer components in Zi-Cao-Cheng-Qi acting on human renal tubular ACHN cell line, hepatoma HepG-2 cell line and HCC HeLa cells [16] and determine major antitumor components of Yinchenhao decoction based on HepG-2 or ACHN cell lines in vitro and in vivo [17]. Rely on our study, chlorogenic acid primarily acts on MCF-7 cell membrane and exhibits a strong binding affinity with EGFR [14].

Research of renal fibrosis confirms the role of 35 natural products in potentially alleviating renal fibrosis, protecting renal structure and enhancing renal function through modulating varying cytokines [18]. However, no effective drugs had been developed from those natural products. *Piper longum* L (Latin name: Piper longum L., alias: Bibb), a perennial shrub or herbaceous vine in the genus Piper, belongs to the piperaceae family, has effects of warming and tonifying the brain and kidney according to the ancient Chinese medicine book “Ben Cao Shi Yi”, hence serving as Chinese medicine for antimicrobial, antiparasitic, anti-inflammatory, antifibrotic, analgesic, antioxidant, and anticarcinogenic treatment [19]. Importantly, many prescriptions of Chinese medicine for chronic kidney disease (CKD), including those for prevention of renal fibrosis, usually contain *Piper longum* L. For example: the *Piper longum* L ingredient appears in Tibetan medicine Anshen pill, Wenshen Jianpi Decoction and Luobufukebiri Pills, etc. Nevertheless, the potential active ingredients in *Piper longum* L remain largely unknown, limiting its applications. Alkaloids, the main active substances of *Piper longum* L, are known to be important antifibrotic active ingredients [20–22]. One of the alkaloidal components, piperlonguminine has been reported to alleviate renal fibrosis [23]. Whether there are other monomer components in *Piper longum* L able to alleviate renal fibrosis needs to be further verified by experimental screening.

**Fig. 1:**
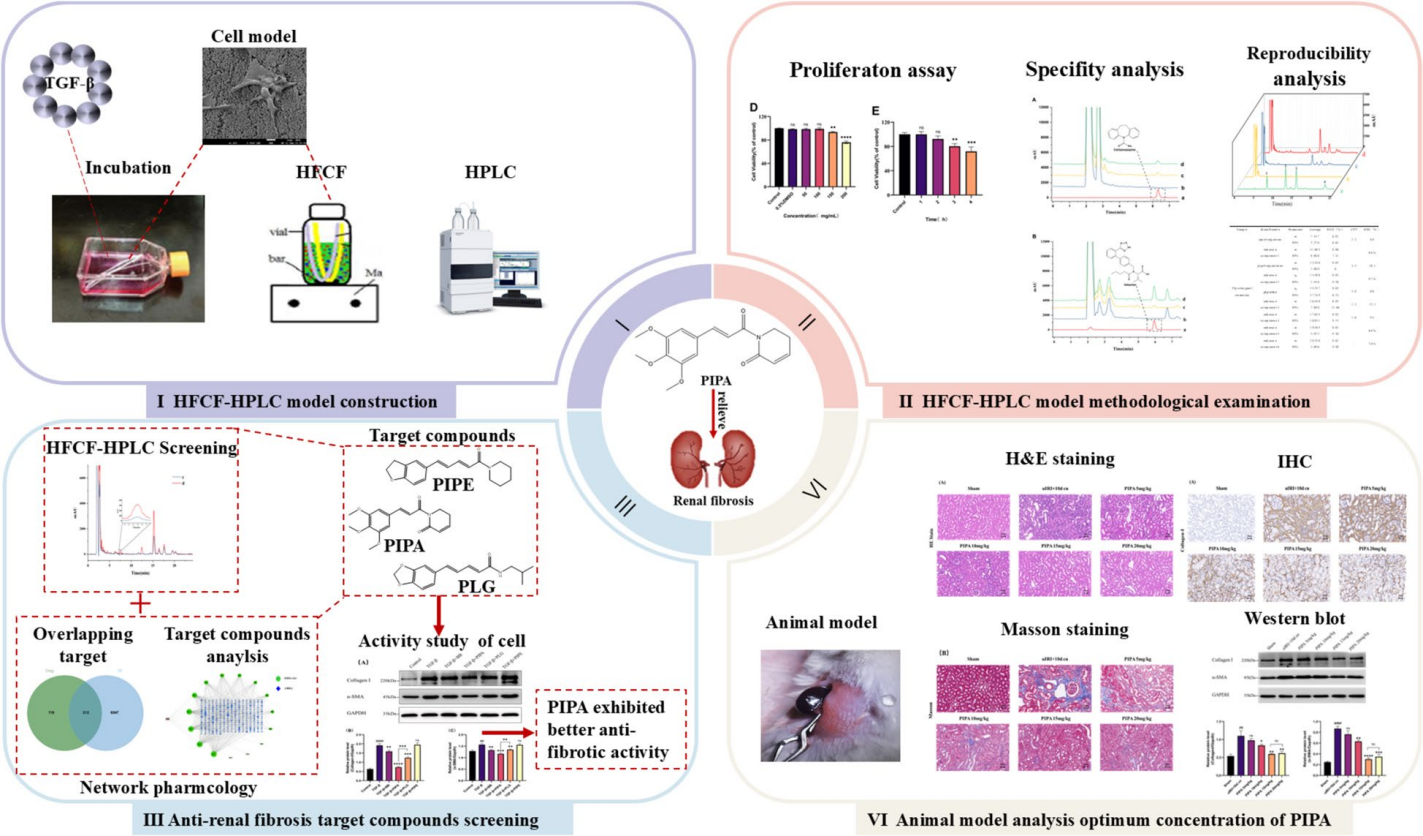
A flow chart illustrated the idea of the study

The summary diagram of this study is shown in Fig. 1. This study established a hollow fiber cell capture model with in vitro renal fibrosis features. It is used in current study combined with the techniques of network pharmacology to screen the active ingredients in *Piper longum* L that are capable of alleviating renal fibrosis. A renal fibrosis cell model and a unilateral nephrectomized ischemia–reperfusion with delayed contralateral nephrectomy animal model (uIRI) were used to validate the pharmacological activity of the compounds with Cell fishing factors (*CFF*, referred to the ratio of the active compound concentration in the hollow fiber with cells (C*cell*) to that in the nutrient hollow fiber (C*nutrient*) following screening balance.) *CFF* > 0. This study developed a new comprehensive system for screening active anti-fibrotic ingredients of traditional Chinese medicines based on HFCF-HPLC combined with network pharmacology and molecular biology, meanwhile added solid scientific evidences for *Piper longum* L being potentially applied to treat renal fibrosis in the future.

## Methods and materials

### Instrument and apparatus

Instruments and apparatus used here included a HPLC (Agilent Technologies, Palo Alto, CA, USA) equipped with two G1311A pumps; a G1316A thermostat, and a VWD UV detector; CO_2_ incubator (Thermo Fisher Scientific, Waltham, MA, USA); a FE-SEM (JSM-7001F, Japan).

HK-2 cell, a human proximal tubular cell line, was provided by the Cell Bank of the Chinese Academy of Sciences (Shanghai, China). DMEM/F12 medium, 10% fetal bovine serum (FBS), 100 U/mL penicillin and 100 μg/mL streptomycin came from Gibco (Grand Island, NY). Trypsin (0.25%, with EDTA), and phosphate-buffered saline (PBS) were purchased from Corning (USA). *Piper longum* L came from Beijing Tongrentang Drugstore (Taiyuan, China). Piperlongumine (PIPA), piperine (PIPE), piperlonguminine (PLG), sesamin, valsartan and carbamazepine (purity > 99%) were purchased from MedChemExpress (USA). Recombinant human TGF-β was provided by R&D Systems (USA). Polyvinylidene fluoride hollow fiber came from Tianjin Motianmo Engineering (Tianjin, China). HPLC-grade methanol and acetic acid glacial were provided by Tianjin Siyou Chemical and Tianjin Guangfu Technology Development (Tianjin, China), respectively. Ultrapure water was obtained by using a Milli-Q A10 water purification system (Millipore, USA). All other reagents were of analytical grade.

Rabbit anti-α-SMA (#19245), anti-GAPDH (#5174) came from Cell Signaling Technology (Danvers, MA, USA). Rabbit anti-collagen I (ab138492) was bought from Abcam (Shanghai, China). Cell counting kit-8 (CCK-8) and RIPA lysis buffer were provided by Wuhan Boster Biological Technology (Wuhan, China). A BCA protein assay kit was provided by Thermo Fisher Scientific (USA). Elisa kit for serum creatinine (SCr) and blood urea nitrogen (BUN) came from Nanjing Jiancheng Biotechnology Research Institute (Nanjing, China).

### Preparation of solution

Standard solutions of PIPA, PIPE, PLG, sesamin, carbamazepine and valsartan (20 mM) were prepared in DMSO. The aliquoted standard solutions were maintained at − 80℃ for subsequent use.

*Piper longum* L was powdered and passed through a 40-mesh sieve, weighed 8 g and placed in a conical flask, then immersed in 160 mL 60% ethanol for 1 h, followed by ultrasonic extraction for 40 min, and filtration. The filtrate was placed in an evaporating dish, evaporated to dry in 60 ℃ water bath to yield the paste, and added with a small amount of DMSO re-soluble. Experimenters diluted the extract to a concentration of 400 mg/mL using F12 complete medium that contained 10% FBS and 1% penicillin/streptomycin (PS) and stored the solution at 4 ℃. The stock solution was diluted to a solution of 100 mg/mL with F12 complete medium (for HFCF-HPLC assay) and filtered with a 0.22 μm microporous filter membrane to acquire the test solution until use (The TCM sample abbreviated as BB).

### Chromatographic condition

An Agilent 1200 series LC instrument (Agilent Technologies, USA) together with ZORBAX Eclipse XDB-C18 column (5 μm, 250 mm × 4.6 mm i.d., Agilent Technologies) served for the chromatographic separation. Mobile phase was adopted with 0.1% acetic acid (A)- Acetonitrile (B) in 45:55 (v/v) (flow rate: 1.0 mL/min, UV absorption: 294 nm, injection volume: 20 μL, and temperature: 40 ℃).

### HK-2 and model cell culture

HK-2 cells underwent culture in DMEM/F-12 added with 10% FBS and 1% PS. The renal fibrosis model of HK-2 cells (model cell) first received 36 h of stimulation by 10 ng TGF-β [24, 25]. Cells were grown in conditions of a humidified atmosphere; 5% CO_2_; a temperature of 37 °C. Digestion began when cells reached 80% confluence.

### Preparation of cell-seeded hollow fiber

Before cell seeding, hollow fibers underwent 10 min of ultrasonication in acetone, methanol and double-distilled water, respectively, to eliminate any possible impurities, followed by air drying. The fibers were then sliced into 7 cm-long segments and underwent high-pressure sterilization. After PBS wash and 2 min of digestion using trypsin at 37 °C, HK-2 cells in logarithmic growth phase received 5 min of centrifugation at 1000 rpm. With the supernatant fluid being eliminated, the F12 complete medium was added to yield a cell suspension, which was injected into the sterilized fiber (length: 7 cm) using a needle-free syringe until another port started to release the cell suspension. A culture flask with the cell-seeded fibers was added with the medium. In the model group, 10 ng TGF-β was added into medium to undergo 36 h of cultivation. With the aim of characterizing the surface features pertaining to cell-seeded or medium seeded fiber, we examined as well as compared their inner surfaces based on the SEM images. The different cell-seeded fibers were removed and placed in 2.5% glutaraldehyde for 4 h, and, after the glutaraldehyde were naturally evaporated, were sliced to make their inner surfaces exposed, followed by SEM photographing at 2.0 kV and 10,00 amplification factors.

### HFCF-HPLC analysis system

The HFCF-HPLC proceeded following previous description [17]. *Piper longum* L sample 6 mL was placed in a 10 mL flatbottom vial containing a stir bar (8 mm × 4 mm) on a magnetic stirrer. The 6 hollow fibers were seeded with HK-2 cells, with both ends sealed with cotton threads, bent into U-shaped, and inserted simultaneously into the above vial that contained *Piper longum* L sample. The hollow fibers underwent 1.5 h of stirring at 600 rpm at 37 °C to screen the active components. Then, experimenters took up the hollow fibers and cut both the sealed ends. The components bound underwent desorption treatment using 30 μL of methanol, followed by being placed into clean and dry Eppendorf tubes (EP, 500 μL). Then, the hollow fiber inner wall was eluted by extracting twice the amount of methanol with a microinjection needle, and such process was conducted twice. The liquids were mixed by 1 min of vortexing and 10 min of centrifugation (10,000 rpm) at 4 °C in succession. The supernatant was moved to a new EP tube and evaporated to dryness, and the residue was reconstituted by adding 30 μL of methanol and then centrifuged again for 5 min after ultrasonication and vortexed for 1 min each. The supernatant was collected for HPLC analysis. Hollow fibers filled with culture medium were used as a blank control group according to the above procedure. The above experiments were performed three times in parallel.

After HFCF-HPLC screening, the ability of the compounds to bind specifically to cells was expressed as *CFF*, *CFF* of the active compounds defined as the ratio of C*cell* to C*nutrient* following screening balance [14]. The formula is as follows:

1 $$CFF=\frac{{C}_{cell}}{{C}_{nutrient}}$$

### Cell proliferation assay

CCK-8 served for the cell proliferation assay as per the producer’s protocol. Briefly, HK-2 cells fell into control group and TCM group with various concentrations (*Piper longum* L sample concentrations: 50, 100, 150, 200 mg/mL), and then, according to the HFCF-HPLC procedure, were treated for varying duration of time. At last, with the CCK-8 reagent being added for 1.5 h, we measured the absorbance at 450 nm. One-way ANOVA assisted in confirming the significant differences.

### Animal experiments

All protocols of animal experiments were approved by the Animal Ethics Committee of the First Hospital of Shanxi Medical University (ethics number: DWYJ-2022-006). Thirty wild type C57BL/6J mice (Academy of Military Medical Sciences, Beijing, China, 7–8 weeks old) were housed in cages with free access to feed and water for a week, followed by being randomized into six groups (n = 5): Sham group (corn oil, 200 μL), uIRI group (corn oil, 200 μL), uIRI + PIPA 5 mg group (PIPA, 5 mg/kg), uIRI + PIPA 10 mg group (PIPA, 10 mg/kg), uIRI + PIPA 15 mg group (PIPA, 15 mg/kg), uIRI + PIPA 20 mg group (PIPA, 20 mg/kg). Mice were administered corn oil and PIPA solution by intra-abdominal injection once a day. All groups were continuously administering corn oil or PIPA solution for 28 days (Fig. S1). After the mice were executed, the injured kidney samples were taken out to undergo fixation in 4% paraformaldehyde, paraffin-embedding and sectioning in succession. Samples were subjected to Masson, H&E, and IHC staining as per the producer’s protocol, with respective images being randomly captured via microscopy. The rest of fresh kidney samples were maintained at − 80 °C.

### Statistical analysis

Data analysis relied on IBM SPSS 22, while GraphPad Prism 8.0 was utilized for graphical representation. Results are in the format of mean ± standard deviation (SD). GraphPad Prism 8 was employed to plot the data. One-way ANOVA served for analyzing the data between multiple groups, and t test compared the data between two groups. *P* < 0.05 indicated statistical significance.

## Results

### Cell growth and survival rate on fiber’s inner wall

**Fig. 2:**
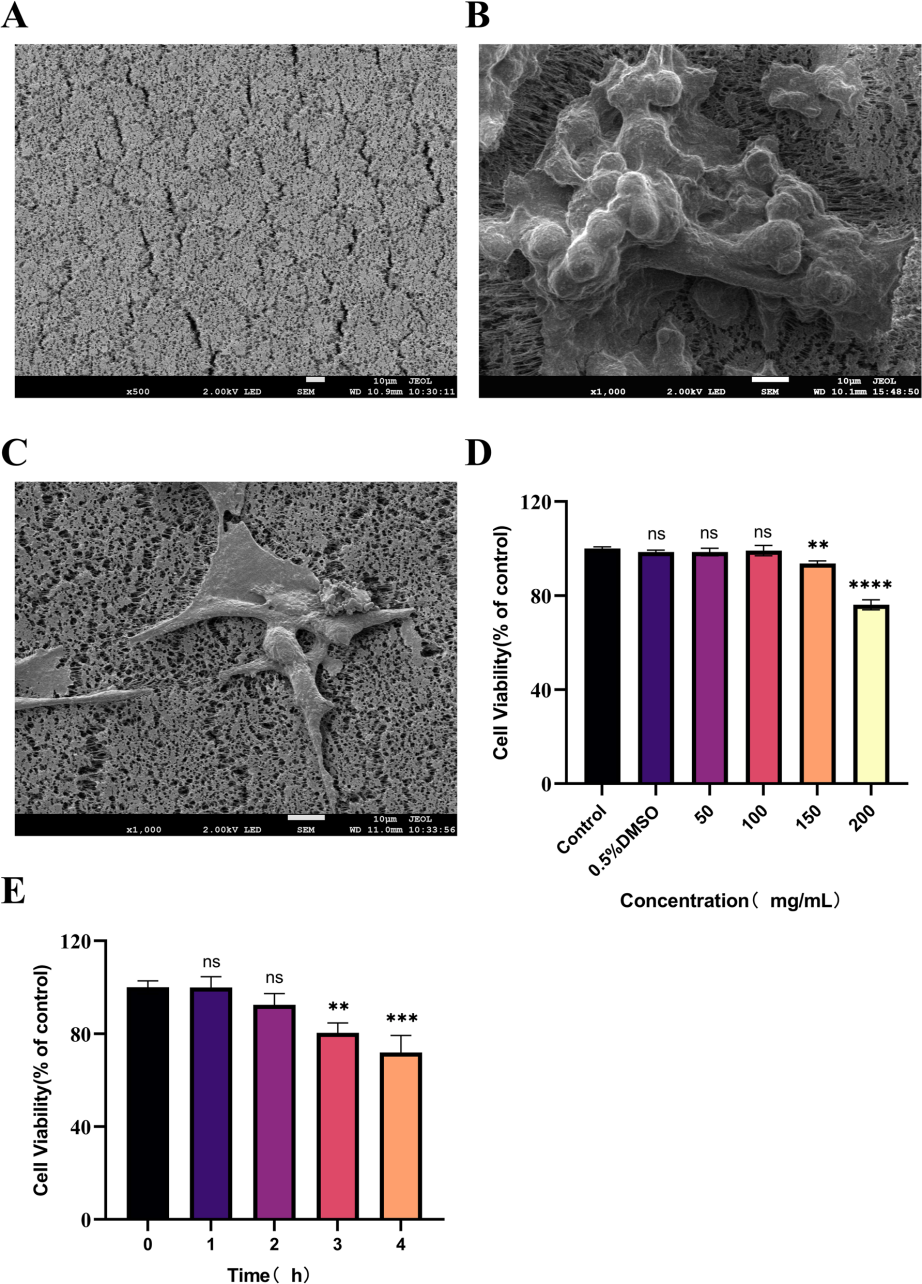
Morphology and proliferative activity of cells seeded in hollow fiber inner surface. **A** SEM micrographs of the inner surface of medium-seeded hollow fiber. **B** HK-2 cell-seeded hollow fiber. **C** Model cell-seeded hollow fiber. **D** The hollow fibers seeded with model cells were inserted into the vial containing *Piper longum* L sample (50, 100, 150, 200 mg/mL) for 1.5 h. **E** The hollow fibers seeded with model cells were inserted into the vial containing *Piper longum* L sample (100 mg/mL) for 1, 2, 3, 4 h. ^*^P < 0.05, ^**^P < 0.01, ^***^P < 0.001, ^****^P < 0.0001 vs Control

With the objective of characterizing the surface features pertaining to cell- or medium-seeded fiber, the study examined as well as compared their inner surfaces according to SEM images. The culture flask with cell-seeded fibers was filled with the medium. The model group added 10 ng TGF-β into medium for induction of fibrosis model of HK-2 cells, and then cultivated for 36 h. Fibers were removed and placed in 2.5% glutaraldehyde for 4 h. After a natural evaporation of the glutaraldehyde, the fibers were sliced to make their inner surfaces exposed, followed by the SEM photographing at 2.0 kV and 10,00 amplification factors. Medium-seeded fiber has uniformed distributed particles in inner wall, together with a smooth plane (Fig. 2A). Distinct bulge can be observed in the inner wall of HK-2 cell-seeded fiber (Fig. 2B), and the cells are morphologically rounded and stacked to cover the fiber surface. Fibrotic cells aggregate and cover the inner wall of model cell-seeded fibers, and a significant change in cell morphology from normal HK-2 cells is observed (Fig. 2C), with a shuttle shaped transformation, and morphological features of fibrosis [26].

The HFCF-HPLC screening process is complex. According to previous research experience, the time required for the active ingredient to pass through the pores of the hollow fiber wall followed by binding to the cells to reach equilibrium is 1.5 h. Consequently, cells must present a good growth along with a high survival rate on hollow fiber inner wall, meanwhile preserving specific cell viability in the screening process. The cell survival rate was significantly reduced after treatment with 150 and 200 mg/mL of *Piper longum* L sample versus the control group (*P* < 0.01) (Fig. 2D). Therefore, to ensure consistent cell activity throughout the screening process, we chose 100 mg/mL as the screening concentration of the Wicker Welfare Extract. In addition, cells exhibited obviously lower survival rate after the screening time was greater than 3 h (Fig. 2E) (*P* < 0.01). Therefore, based on the above experimental results, a concentration of 100 mg/mL of *Piper longum* L sample can ensure the result reliability.

### Reliability study of the HFCF-HPLC model

**Fig. 3:**
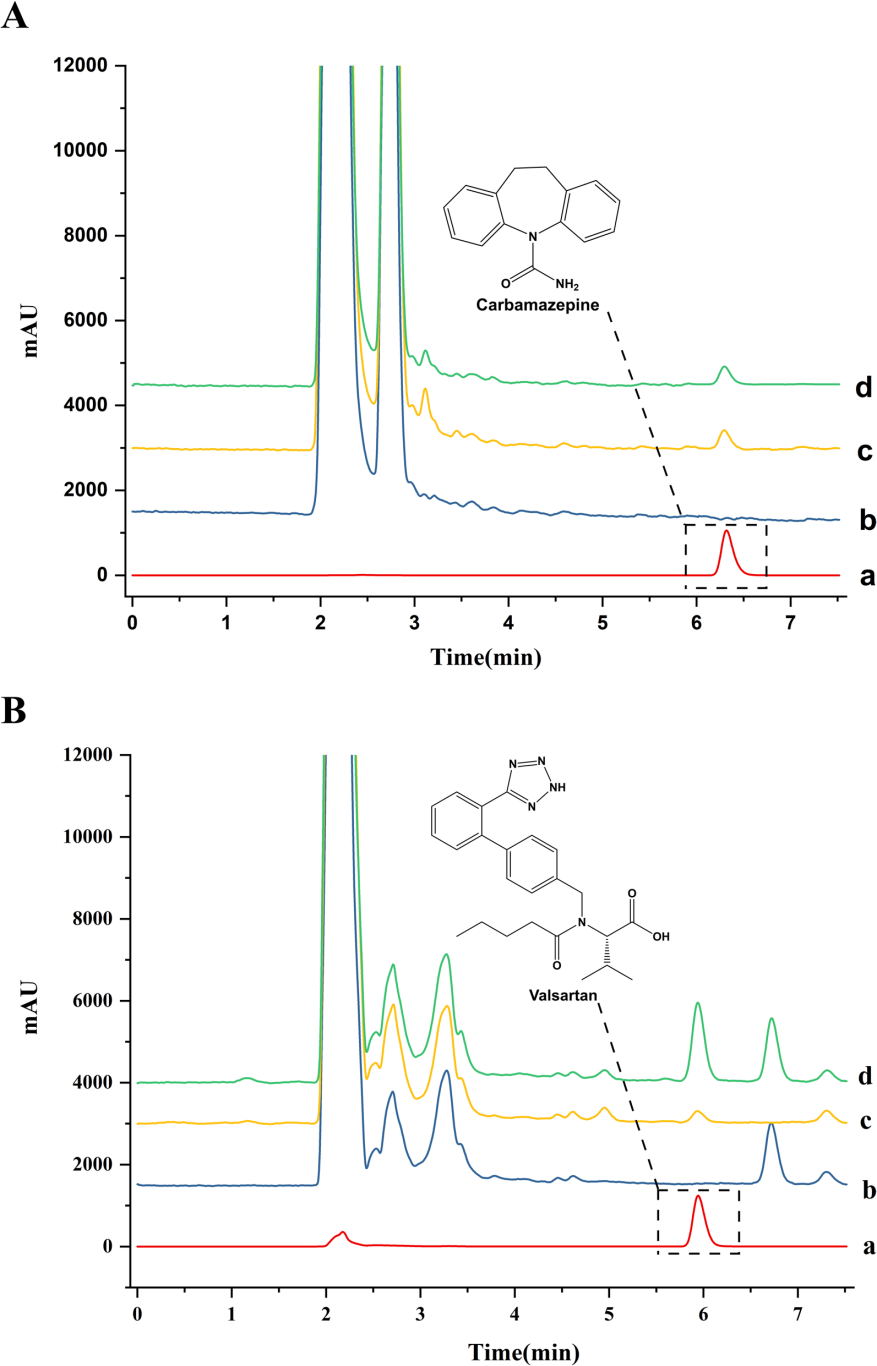
Chromatograms of Carbamazepine fished by model cells (**A**) and Valsartan by model cells (**B**). a, standard solution; b, blank control; using hollow fiber with: c, nutrient solution (DMEM/F-12 containing 10% FBS); d, model cells

To validate the HFCF-HPLC reliability, we have selected valsartan and carbamazepine as positive and negative controls, respectively, for the reason that valsartan can improve the prognosis of patients with CKD [27], and no relevant studies have shown an ameliorative effect of carbamazepine on kidney disease. *CFF* as the index used to analyze cell-drug binding ability. After screening by HFCF-HPLC (Fig. 3A and B), the negative control drug carbamazepine retention times (*t*_R_) = 6.4 with *CFF* ≈ 0, while the positive control drug valsartan *t*_R_ = 6.0, active component screened by hollow fiber seed cells demonstrated a remarkably higher concentration versus that screened by hollow fiber with medium, with *CFF* = 5.3. Therefore, the specific binding ability of valsartan to model cells was significantly higher than that of carbamazepine, indicating that HFCF-HPLC has good specificity and can serve for comprehensively screening active compounds in complicated systems.

Whether the screening results are reproducible greatly affects the method reliability. When using HFCF-HPLC to screen the active components in the BB and biological samples, we conducted three times of parallel determination on their *t*_R_ and relative peak area (RPA) (Table 1). The relative standard deviations (RSD) of the t_R_ (n = 3) for each active ingredient were in the range of 0.01–0.07%, and the RSD values of RPA (n = 3) were in the range of 7.31%−11.66%. Therefore, the reproducibility of the HFCF-HPLC method established in this study for the screening of alternative components in *Piper longum* L was high.

**Table 1:** The key active substances in the “*Piper longum* L compound-target-renal fibrosis (RF)” network

No	Mol ID	Name	OB	DL	OD
BB1	MOL001555	diaeudesmin	52.35	0.62	54
BB2	MOL001558	piperlonguminine	56.55	0.83	29
BB3	MOL001559	sesamin	30.71	0.18	80
BB4	MOL001560	pipernonaline	51.32	0.41	32
BB5	MOL001561	dehydropipernonaline	47.73	0.41	33
BB6	MOL001586	N-(2,5-dimethoxyphenyl)−4-methoxybenzamide	60.7	0.18	93
BB7	MOL001592	piperine	42.52	0.23	99
BB8	MOL001594	pisatin	88.05	0.64	51
BB9	MOL001601	tetrahydrotanshinone I	38.75	0.36	43
BB10	MOL001607	ZINC03982454	36.91	0.76	1
BB11	MOL001610	sylvatine	44	0.51	2
BB12	MOL001614	Retrofractamide B	42.72	0.43	11
BB13	MOL001616	1-[1-oxo-9(3, 4-methylenedioxyphenyl)−2E,8E-nonadienyl] pyrrolidine	49.43	0.36	96
BB14	MOL002848	piperlonguminine	96.65	0.24	89
BB15	MOL002857	55,038–30-7	42.64	0.53	1

### Active ingredient screening

According to the results of web pharmacology prediction (Fig. S2A and B), we sorted all chemical components by their degree, and confirmed the key active substances for *Piper longum* L in treating renal fibrosis (Table 1), in which the content of tetrahydrotanshinone I and 4-methoxy-N- (2,5-dimethoxyphenyl) benzoyl in the extraction of *Piper longum* L were very low, and couldn’t be detected in the chromatogram (below detection limit). In contrast, pipernonaline is a hazardous material with a Hazard Category Identification Number of 22, suggesting toxicity if swallowed (as shown on pubchem), therefore unsuitable for further experimental studies. On this basis, combined with the results of studies related active ingredients of traditional Chinese medicine alleviated renal fibrosis [23, 28], PIPE, sesamin, PIPA and PLG were selected to configure standard solutions for HPLC analysis, which were used to identify the active ingredients obtained from the HFCF-HPLC screening.

**Fig. 4:**
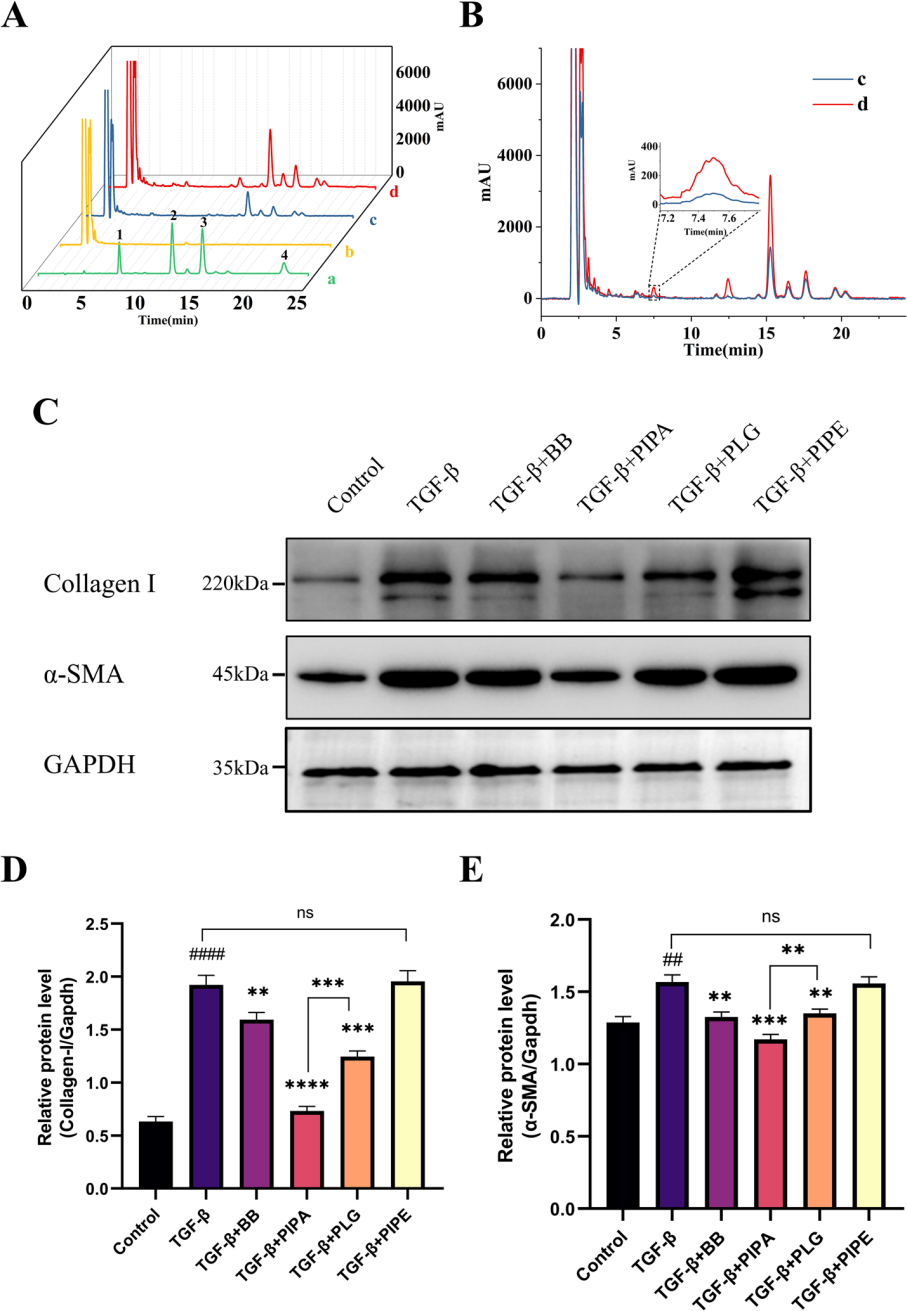
Target identification based on HFCF-HPLC and in vitro experimentation. **A** Chromatograms of the active compounds screened by model cells. **B** Superimposed chromatograms of c and d.; a, standard solution; b, blank control; using hollow fiber with: c, nutrient solution (DMEM/F-12 containing 10% FBS); d, model cells; **C** Western blots of fibrosis markers after 36 h of incubation using different components. Quantitative outcome of expression levels of **D** Collagen-I, and **E** α-SMA. ^#^*P* < 0.05, ^##^*P* < 0.01, ^###^*P* < 0.001, ^####^*P* < 0.001 versus the control group; ^*^P < 0.05, ^**^P < 0.01, ^***^P < 0.001, ^****^P < 0.0001 versus the TGF-β group for (**D**, **E**)

The HFCF-HPLC model engaged in screening the alternative components for alleviating renal fibrosis in the extract of *Piper longum* L (Table 2; Fig. 4A). There was a total of 9 distinct peaks in the chromatogram, including 3 known components and 6 unknown components. (1) The chromatogram did not show any peaks of sesamin, probably the content of sesamin in the extract was lower than the detection limit. (2) Three alternative anti-renal fibrosis components were identified based on the retention time of the standard (*CFF* > 0), including: PIPA (*t*_R_ = 7.537), PLG (*t*_R_ = 12.448), and PIPE (*t*_R_ = 15.257) (Fig. 4B); (3) Two unknown components were also identified with *CFF* > 0 during the screening process, unknown component 3 (*t*_R_ = 16.450) and unknown component 4 (*t*_R_ = 17.635). (4) In addition, there were two other unknown components with *CFF* ≈ 0 in the chromatogram, unknown component 5 (*t*_R_ = 19.583) and unknown component 6 (*t*_R_ = 20.258), which were not reflected in Table 1. The results of this experiment suggest that there are five alternative components in *Piper longum* L that can specifically bind to model cells, and thus the component that exerts anti-fibrotic effects may not be a single component.

**Table 2:** Reproducibility of HFCF-HPLC and screened active components (n = 3)

Sample	Identification	Parameter	Average	RSD(%)	*CFF*	RSD(%)
*Piper longum* L extraction	Piperlongumine	*t*_R_	7.537	0.07	2.3	8.9
		RPA	2.228	8.61		
	Unknown component 1	*t*_R_	11.682	0.06	-	8.6%
		RPA	0.800	7.31		
	Piperlonguminine	*t*_R_	12.448	0.03	3.4	10.1
		RPA	1.000	0		
	Unknown component 2	*t*_R_	14.508	0.03	-	9.7%
		RPA	1.550	9.20		
	Piperine	*t*_R_	15.257	0.03	3.8	6.6
		RPA	27.249	8.24		
	Unknown component 3	*t*_R_	16.450	0.02	1.3	12.1
		RPA	7.088	11.66		
	Unknown component 4	*t*_R_	17.635	0.03	1.6	9.4
		RPA	10.031	9.13		
	Unknown component 5	*t*_R_	19.585	0.01	-	6.8%
		RPA	3.922	9.30		
	Unknown component 6	*t*_R_	20.258	0.01	-	7.8%
		RPA	3.006	9.90		

*RPA* Ratio of the peak area of each active component to that of piperlonguminine, *CFF* C*cell*/C*nutrient*

### Anti-fibrotic activity of major active compounds in vitro

Integrating the combined network pharmacology and HFCF-HPLC model, we further validated the cellular-level bioactivities of *Piper longum* L extraction (BB), PIPA, PLG, and PIPE. The study set the intervention concentrations of PIPA and PLG at 10 μmol/L and PIPE at 20 μmol/L according to the literature. 100 mg/mL of BB was selected according to the results of the cellular CCK-8 experiment. The model group exhibited obviously higher expressions of α-SMA (*P* < 0.05) and Collagen-I (*P* < 0.0001) versus the control group, with the difference showing statistical significance (Fig. 4C–E); whereas BB, PIPA and PLG all had different effects in decreasing the expression of Collagen-I (Fig. 4D) and α-SMA (Fig. 4E), with PIPA being the most effective, with a statistically significant difference (*P* < 0.001) versus the TGF-β group. PIPE basically exerted no impact on α-SMA and Collagen-I expressions, and no significant difference was observed versus the TGF-β group (*P* > 0.05). The active components that exerted anti-fibrotic effects were PIPA and PLG. PLG had been reported to alleviate renal fibrosis [23], consistent with our observations (Fig. 4C–E). Nevertheless, PIPA exhibited better anti-fibrotic activity against renal fibroblasts according to its inhibitory mechanism against α-SMA and Collagen-I at the cellular level.

### PIPA alleviated renal interstitial fibrosis in vivo

**Fig. 5:**
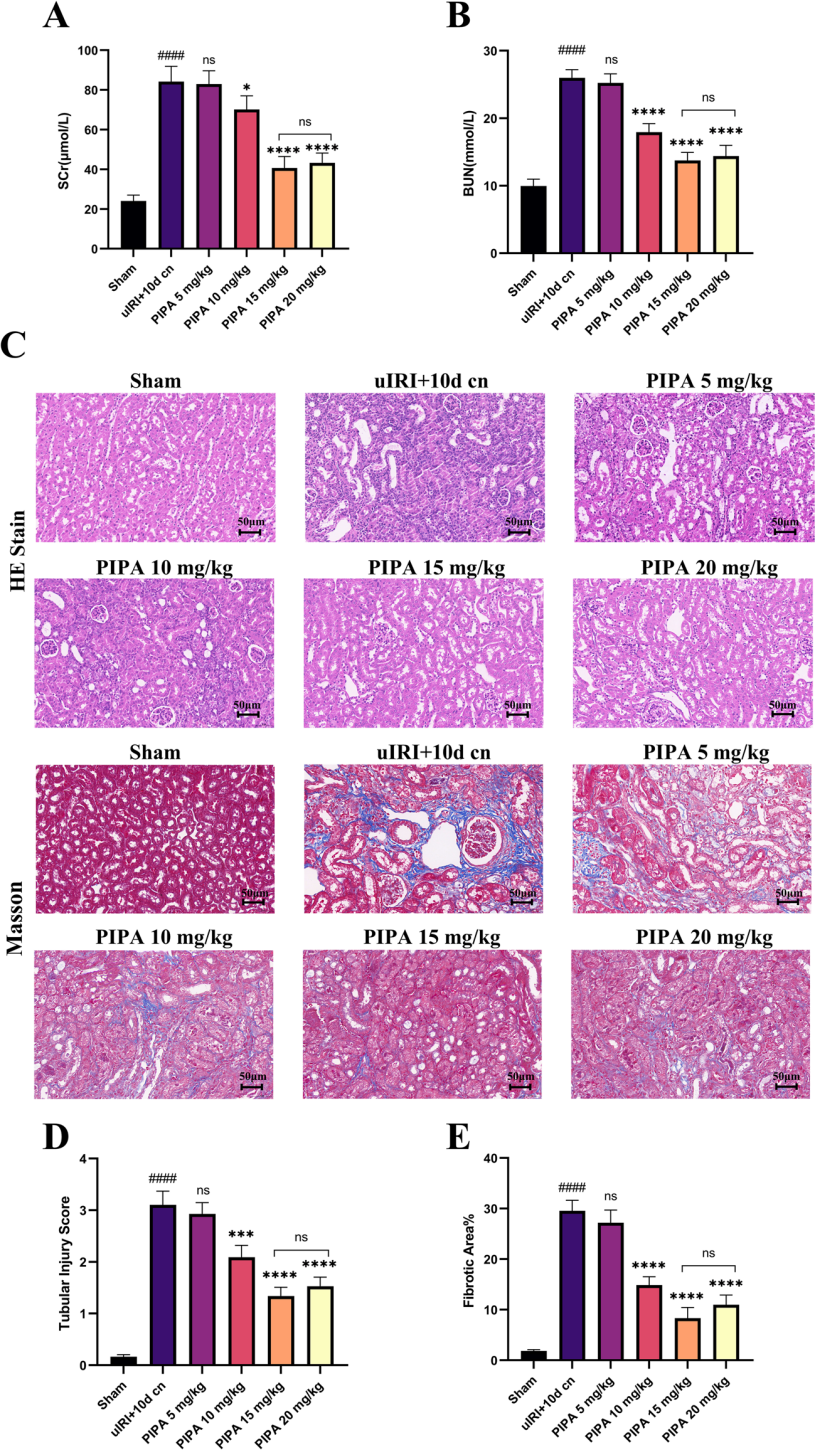
PIPA weakened I/R-induced mice renal injury and fibrosis. The right kidney and the insulted left kidney were harvested on day 10 and 28, respectively, after I/R injury. **A**, **B** Kidney function of left kidney. **C–E** Representative images of H*&*E and Masson (scale bar = 50 μm)

A uIRI (left kidney) of renal fibrosis model with delayed contralateral nephrectomy (right kidney) was performed to elucidate the pharmacodynamic effects of PIPA in vivo. According to the testing results of renal function, the left kidneys suffered serious kidney injury, while such injury could be alleviated by PIPA. uIRI + delayed contralateral nephrectomy model group showed much higher SCr and BUN versus the sham group (*P* < 0.0001) (Fig. 5A and 5B). Mice receiving ≥ 10 mg/kg PIPA exhibited obviously lower BUN and SCr versus the model group, with 15 mg/kg being the most effective dosage (*P* < 0.0001).

**Fig. 6:**
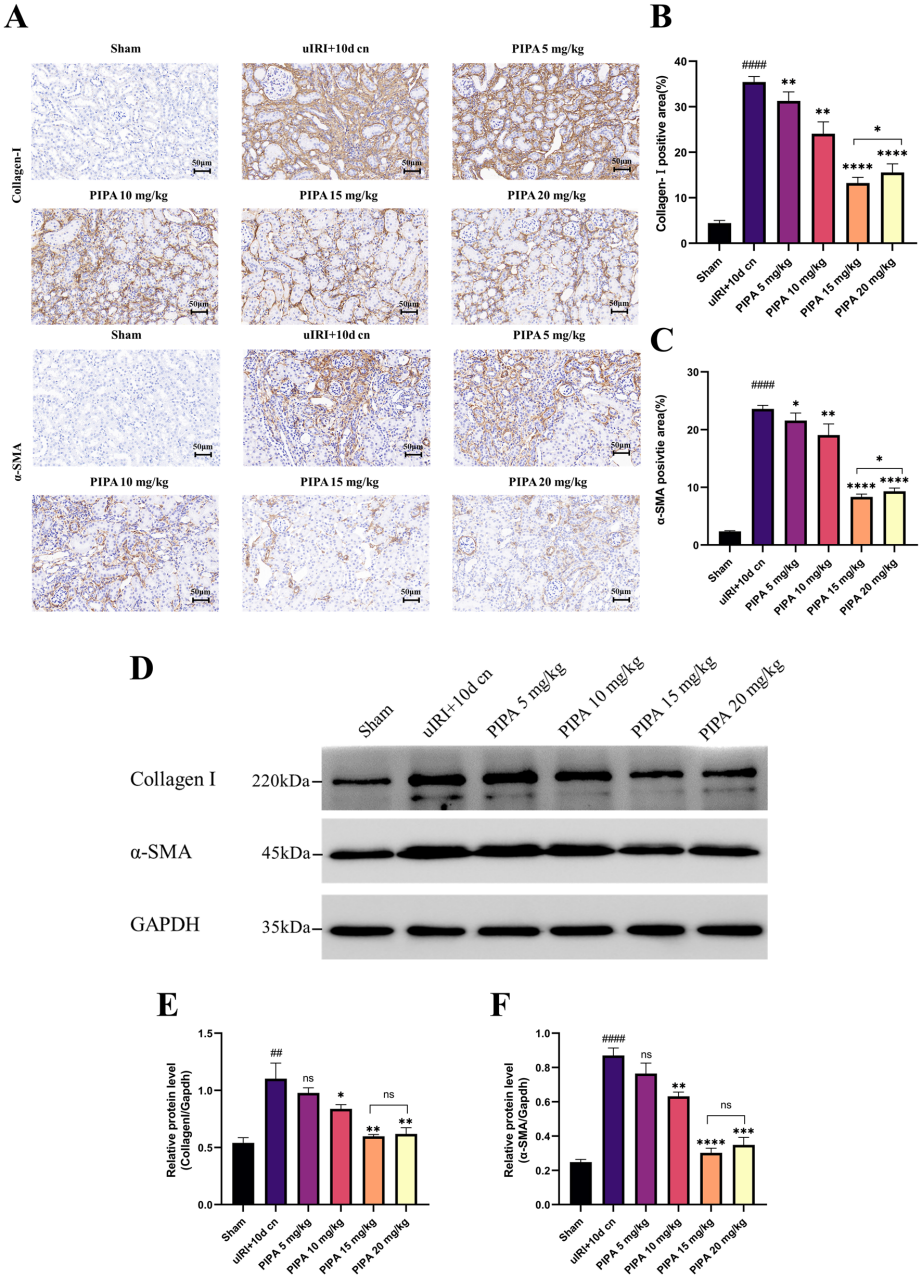
PIPA weakened I/R-induced mice renal injury and fibrosis. **A–C** Representative images of IHC staining: α-SMA and Collagen-I. **D–F** Western blot of fibrosis marker expression. ^#^*P* < 0.05, ^##^*P* < 0.01, ^###^*P* < 0.001, ^####^*P* < 0.001 versus the sham group. ^*^P < 0.05, ^**^P < 0.01, ^***^P < 0.001, ^****^P < 0.0001 versus the uIRI +  10 d cn group for (**A–D**)

Four weeks following reperfusion, in relative to the sham group, the model group presented more obvious tubular injury and fibrosis (*P* < 0.0001) under HE staining, with tubules dilation, tubular disorganization, and ECM deposition. Intraperitoneal injection of PIPA weakened renal fibrosis 28 days after I/R injury. Kidney pathology demonstrated reduced injury and fibrosis in macroscopical level, and the degree of reduction in fibrosis changed with the rising PIPA concentrations. The intervention group exhibited remarkably lower Masson (mainly delineating the fibrosis), particularly in model + 15 mg/kg group (*P* < 0.0001) (Fig. 5C–E). Besides, according to IHC staining, mice receiving ≥ 15 mg/kg PIPA showed remarkably lower a-SMA and collagen-1 expressions in the interstitial area versus the model group on D 28 (*P* < 0.0001) (Fig. 6A–C). To further corroborate this, we conducted western blotting (Fig. 6D–F), confirming that these fibrosis markers changed in accordance with the histopathological variation. Collectively, PIPA had a therapeutic effect in reducing renal fibrosis.

## Discussion

There is still no proven treatment for renal fibrosis [29], although many therapeutic attempts have been made [30]. Many proprietary Chinese medicines containing *Piper longum* L for renal function regulation can alleviate the progression of CKD, and the alkaloid component PLG in *Piper longum* L has been reported to be able to alleviate renal fibrosis [23]. Nevertheless, researchers encounter difficulties in well elucidating the Chinese herbal formula’s complex composition, making the articulation of the active ingredients a key to achieving TCM modernization and globalization.

In this study, the HFCF-HPLC model was reconstructed, and its specificity, reproducibility, and applicability were evaluated. This novel model screened the anti-renal fibrosis active ingredients of *Piper longum* L. Compared with the previous HFCF-HPCL model, some major improvements were made in this study are as follows. (1) The type of media fiber was optimized as PP (Polypropylene) hollow fibers, to avoid the screening result being interfered by non-specific binding, instead of the previous PVDF (Polyvinylidene fluoride) hollow fiber containing a large number of fluorine atoms, which is rich in electrons highly susceptible to the formation of charge transfer supramolecules with some components of the herbal extracts [31]. (2) This study reconstructed the HFCF-HPLC model by inducing HK-2 cells to undergo fibrosis, which is applicable not only to the screening of anti-tumor active ingredients but also to that of anti-renal fibrosis active ingredients, thereby significantly broadening the scope of application of the model. (3) In this study, complete medium was used to dissolve the extraction of *Piper longum* L to ensure that the cells could obtain nutrients during the screening process and maintain a suitable osmotic pressure to prevent the occurrence of cell lysis, resulting in the cells of the fiber inner wall achieving a high survival rate throughout the screening process, which provides the stability of screening results shown in the cell survival results (Fig. 2D, E). Nevertheless the RSD values of the repeatability data for the HFCF-HPLC peak areas were relatively high (Table 2), albeit within the acceptable range. The reasons may be: (1) very low concentration of the test samples, usually ranging from tens to hundreds of ng; (2) the complexity of the steps of the HFCF-HPLC operation process, in which multiple factors including sample preparation, experimental operations, and cell culture conditions, etc., may collectively influence the results. For future research, a series of corresponding improvement measures will be explored to guarantee that the screening results are accurate and reliable.

This study applied a novel comprehensive screening strategy combined with the HFCF-HPLC model, network pharmacology, and molecular biology techniques to improve screening efficiency and accuracy. The theoretical bases of the HFCF-HPLC model and network pharmacology are different. The HFCF-HPLC method, which uses living cells to screen the herbal extracts, allows screening for constituents with lower binding capacity, higher content, and high cell contact through the fiber wall pores, such as PLG (degree = 29). In contrast, network pharmacology, as a predictive method based on database and software analysis, can predict some compounds present at very low amounts in the herbs but with high binding to the disease target, such as tetrahydrotanshinone I and 4-methoxy-N-(2,5-dimethoxyphenyl) benzoyl. As is shown in Tables 1 and 2, the results of the HFCF-HPLC model screening for the anti-renal fibrosis active ingredients of *Piper longum* L did not exactly match with those of network pharmacology. (1) HFCF-HPLC screening detected PIPE, PIPA and PLG with the *CFF* values of 3.8, 2.3 and 3.4, and no sesamin for its low content in the extracts of *Piper longum* L. (2) The predicted degrees of PIPE, PIPA, PLG and sesamin from the network pharmacology results were 99, 89, 29 and 80, respectively. The results of further cell viability experiments showed that both PIPA and PLG in *Piper longum* L had an alleviating effect on renal fibrosis, and PIPA was more effective (Fig. 4C, D). Therefore, the screening strategy applied in this study can provide more comprehensive and accurate results.

With the aim of more deeply validating the anti-renal fibrosis activity of PIPA, we conducted in vivo studies by selecting the uIRI model with delayed contralateral nephrectomy in C57BL/6J mice, which more closely resembles the actual pathological process caused by acute kidney injury, ie, from nephritis, renal fibrosis, CKD to end-stage renal disease [32]. These results indicate the comprehensive strategy based on the HFCF-HPLC model, network pharmacology, and molecular biology techniques was efficient, comprehensive and accurate for screening anti-renal fibrosis active ingredients of the herbs.

Renal fibrosis is a complicated process that involves varying cell types, signaling pathways, and metabolic alterations. Angiotensin receptor blockers (ARBs) like valsartan can effectively reduce urinary protein of CKD patients, but are incapable of reversing their renal fibrosis. Besides, the side effect of ARBs is serious reduction of the patients’ life quality [33]. Therefore, further research for more effective and safer therapeutic agents is urgently needed. This study confirmed the antifibrotic effect of PIPA both in vitro and in vivo, and it has multiple molecular effects closely related to the alleviation of fibrosis, among which two of the important effects are as follows. (1) PIPA has an inhibitory effect on TGF-β induced epithelial-to-mesenchymal transition (EMT) through regulating the expressions of E-cadherin, Snail1, and Twist1[34]. It has long been recognized that EMT is a key mechanism to promote renal fibrosis, and effective inhibition of EMT can significantly alleviate fibrosis [35]. (2) PIPA promotes the expression of ICAT by upregulating LINC01391 [36]. Meanwhile, ICAT can inhibit β-catenin/TCF1 complex from being formed, promote the increase of β-catenin/FoXO1 complex ratio, thereby leading to decrease of TF ratio, and exert a good antifibrotic effect [37]. According to the experimental results combined with the above theories, PIPA may regulate and alleviate renal fibrosis through multiple pathways, which is conducive to the development of antifibrotic drugs with high efficacy and safety.

## Conclusions

The present study constructed a novel comprehensive strategy to screen the active ingredients in the *Piper longum* L for alleviated kidney fibrosis: network pharmacology for ingredients forecasting, HFCF-HPLC model for compound fishing, and molecular biology techniques for efficacy verification. The following conclusions were obtained: (1) A fast and reliable HFCF based on HK-2 cells induced by TGF-β coupled with HPLC was first developed by us as a pre-liminary drug screening tool for researching major anti-fibrotic active components of *Piper longum* L, and potentially for further expansion of HFCF-HPLC in screening other active ingredients. (2) Our results confirmed PIPA as another anti-fibrotic alkaloid active ingredient in *Piper longum* L and experimentally assisted researchers in enhancing its anti-fibrotic applications.

## Supplementary Information

Additional file 1

## Data Availability

All data generated or analyzed herein can be found in this published article and its supplementary information files.

## Abbreviations

TCM: Traditional Chinese medicine
CKD: Chronic kidney disease
HFCF: Hollow fiber cell fishing
HPLC: High-performance liquid chromatography
TGF-β: Transforming growth factor-β
uIRI: Unilateral ischemia–reperfusion injury
SCr: Serum creatinine
BUN: Blood urea nitrogen
CMC: Cell membrane chromatography
CFF: Cell fishing factors
FBS: Fetal bovine serum
PBS: Phosphate-buffered saline
PIPA: Piperlongumine
PIPE: Piperine
PLG: Piperlonguminine
CCK-8: Cell counting kit-8
PS: Penicillin/streptomycin
TR: Retention times
RPA: Relative peak area
RSD: Relative standard deviations
AKI: Acute kidney injury
EMT: Epithelial to mesenchymal transition
ICAT: Inhibitor of β-catenin and T-cell factor
TCF1: T cell factor 1
FoxO1: Forkhead box O 1
TF ratio: Relative binding of β-catenin/TCF1 versus β-catenin/FoxO1
