# Supplementary material for: Identification of anti-fibrotic compounds from Piper longum L via hollow fiber cell fishing and high-performance liquid chromatography with in vivo and in vitro validation
Source: Chin Med. 2025 Aug 19;20:127. doi: 10.1186/s13020-025-01177-z (PMC12362919; doi:10.1186/s13020-025-01177-z)
Supplement: Supplementary file 1 — Additional file 1 [file 13020_2025_1177_MOESM1_ESM.docx]

**Supplementary Figures**


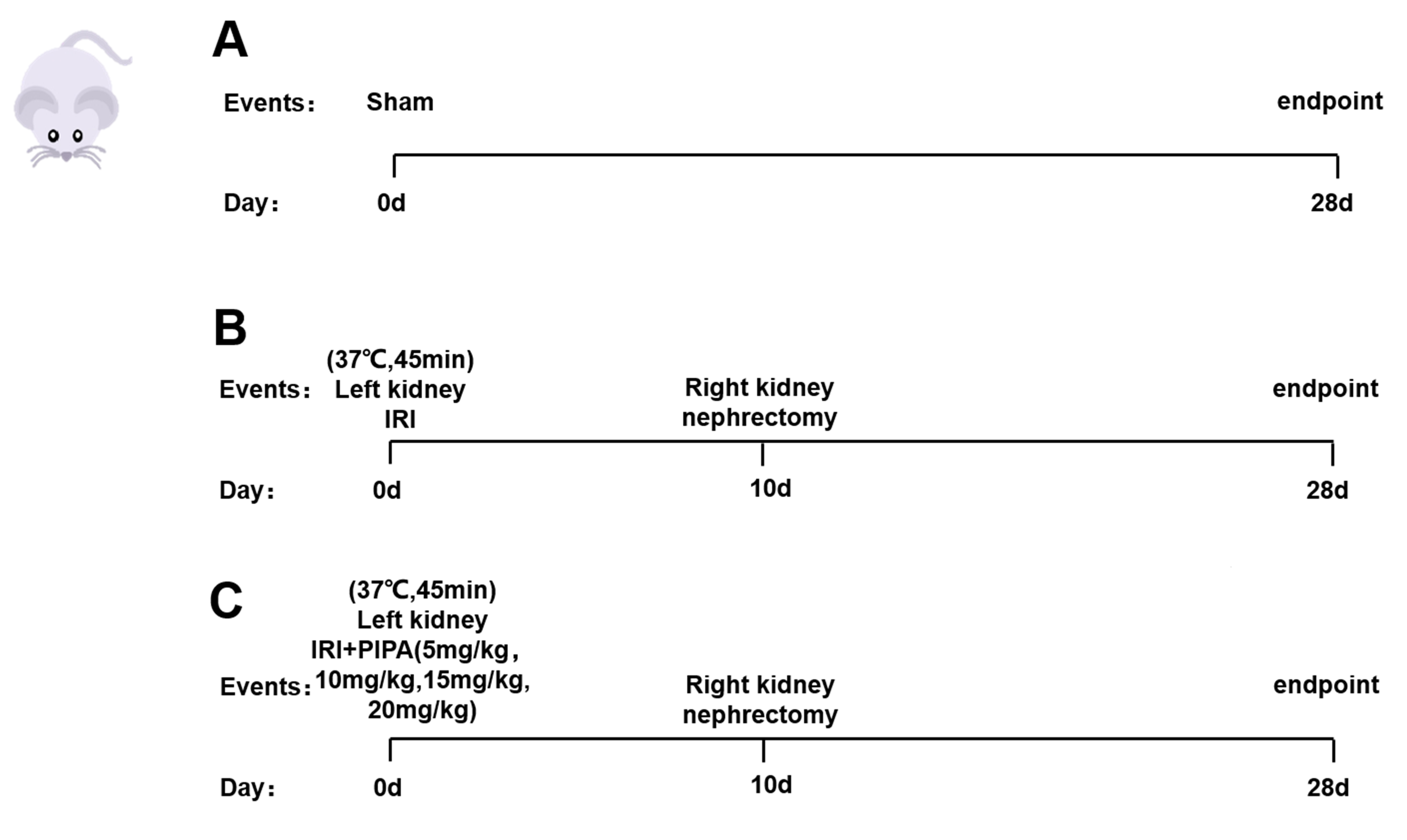


**Fig. S1 Schematic diagram of the animal experiment protocol.**





**Fig. S2 Target identification based on network pharmacology. (A)** Intersection of Renal fibrosis disease targets (RF) and Piperlonguml (Drug) targets. **(B)** Herba Piperlonguml chemical compound-target-RF network.
